# Supplementary material for: Real World Experience of Second-Line Treatment Strategies after Palbociclib and Letrozole: Overall Survival in Metastatic Hormone Receptor-Positive Human Epidermal Growth Factor Receptor 2-Negative Breast Cancer
Source: Cancers (Basel). 2023 Jun 30;15(13):3431. doi: 10.3390/cancers15133431 (PMC10341332; doi:10.3390/cancers15133431)
Supplement: Supplementary file 1 [file cancers-15-03431-s001.zip › cancers-2451410-supplementary.pdf]

Supplementary Table S1. Baseline patient characteristics (N=305)

Characteristic	Progression (N=181)	No progression (N=124)	P-value
Median FU (IQR)	39.8 (30.7,49.7)	44.7 (36.1, 50.1)	
Age			.308
Median (range)	51.5 (31.5, 78.1)	52.0 (33.3, 86.7)	
<50 years old	80 (44.2)	50 (40.3)	
>50 years old	101 (55.8)	74 (59.7)	
ECOG PS[1]			.465
0	104 (57.5)	73 (58.9)	
1	75 (41.4)	47 (37.9)	
≥2	2 (1.1)	3 (2.4)	
Unknown	0	1 (0.8)	
Disease status			.716
De novo	62 (34.3)	45 (36.3)	
Recurred	119 (65.7)	79 (63.7)	
Adjuvant CTx.	92 (50.8)	71 (57.3)	
Neoadjuvant CTx	27 (14.9)	8 (6.4)	
Disease-free interval[2]	(n=198)		.056
<12 months	76 (63.9)	39 (49.4)	
≥12 months	43 (36.1)	40 (50.6)	
Metastatic sites			<.001
Visceral	52 (28.7)	14 (11.3)	
Liver	48 (26.5)	12(9.7)	
Non-visceral	129 (71.3)	110 (88.7)	
Bone only	57 (31.5)	46 (37.1)	
No. of disease sites			.012
1	81 (44.8)	77 (62.1)	
2	68 (37.6)	32 (25.8)	
3 or more	32 (17.6)	15 (12.1)	
Germline BRCA status			.041
Not tested	115 (63.5)	91 (73.4)	
BRCA1/2 mutation	6 (3.3)	0	
No BRCA mutation	60 (33.2)	33 (26.6)	
Initial CA-15-3 status			.001
Normal range	91 (50.3)	85 (68.5)	
Elevated	81 (44.8)	30 (24.2)	
Unknown	9 (4.9)	9 (7.3)	
Initial CEA status			<0.001
Normal range	128 (70.7)	103 (83.1)	
Elevated	49 (27.1)	13 (10.5)	
Unknown	4 (2.2)	8 (6.4)	

1: performance status; 2: defined as breast cancer recurrence within 1 year following adjuvant ET completion

Supplementary Table S2. Second line treatment strategy after palbociclib with letrozole treatment (N=166)

Regimen	N (%)
Capecitabine	47 (28.3%)
Exemestane with everolimus	45 (27.1%)
Cytotoxic chemotherapy except capecitabine	33 (19.8%)
Taxene (paclitaxel, docetaxel)	28 (16.8%)
Anthracycline based regimen	3 (1.8%)
Others*	2 (1.2%)
Fulvestrant, other ET only	23 (13.8%)
Fulvestrant	19 (11.4%)
Palbociclib + fulvestrant	2 (1.2%)
Exemestane	1 (0.6%)
Tamoxifen	1 (0.6%)
Clinical trials	18 (10.8%)

Supplementary Table S3. Disease progression sites after palbociclib with letrozole (N=164)

Organ	N (%)
Visceral	64 (39.0%)
Liver	61 (37.2%)
Central nerve system	4 (2.4%)
Lymphangitic lung metastases	1 (0.6%)
Non-visceral metastasis	53 (32.3%)
Lung	11 (6.7%)
Lymph nodes	27 (16.5%)
Breast	12 (7.3%)
Pleura	8 (4.9%)
Ovary	1 (0.6%)
Stomach	1 (0.6%)
Bone only	47 (28.7%)
